# Donanemab Infusion and Recurrent Seizures in a Patient With Alzheimer’s Disease and Epilepsy in the Absence of Amyloid-Related Imaging Abnormalities (ARIA)

**DOI:** 10.7759/cureus.111911

**Published:** 2026-07-01

**Authors:** Patricio S Espinosa, Sarah Li, Johanna Preisler, Charles H Hennekens

**Affiliations:** 1 Neurology, Espinosa Neuroscience Institute, Boca Raton Regional Hospital, Boca Raton, USA; 2 College of Medicine, Florida Atlantic University, Boca Raton, USA; 3 Neurology, Espinosa Neuroscience Institute, Boca Raton, USA; 4 Preventive Medicine/Cardiology/Internal Medicine, Charles E. Schmidt College of Medicine, Florida Atlantic University, Boca Raton, USA

**Keywords:** alzheimer’s disease, donanemab, epileptic seizures, grand mal seizures, mild cognitive impairment (mci)

## Abstract

Donanemab is an immunoglobulin G1 (IgG1) monoclonal antibody that was recently approved by the United States Food and Drug Administration for the treatment of early symptomatic Alzheimer’s disease (AD). The drug was approved with boxed warnings for amyloid-related imaging abnormalities (ARIA). While seizures are recognized adverse events, they are predominantly characterized in the context of ARIA and accompanied by edema (ARIA-E) or hemosiderin deposition (ARIA-H). The precise epileptogenic mechanisms of anti-amyloid immunotherapies independent of macroscopic ARIA remain poorly understood.

We report the case of a 90-year-old man with mild cognitive impairment due to AD and a history of well-controlled focal epilepsy on levetiracetam, who experienced two generalized tonic-clonic seizures. Each paroxysmal event occurred within two hours of completing his fifth and sixth monthly donanemab (1,400 mg intravenous) infusions. Inter-ictal magnetic resonance imaging (MRI), including fluid-attenuated inversion recovery (FLAIR) and susceptibility-weighted imaging (SWI) sequences, demonstrated no evidence of ARIA-E or ARIA-H. Routine electroencephalography (EEG) revealed no interictal epileptiform discharges. Seizure recurrence necessitated an escalation of his levetiracetam dosage to 750 mg twice daily and the immediate discontinuation of donanemab therapy, after which he remained seizure-free.

The temporal proximity of the seizures to the infusions, the positive rechallenge, and the absence of identifiable structural, metabolic, or infectious precipitants raise the possibility of an association with donanemab. However, causality cannot be established from a single case report. Several biologically plausible mechanisms may explain this temporal association, including rapid amyloid-beta (Aβ) clearance, microglial activation, neuroinflammatory responses, and transient blood-brain barrier dysfunction, which may lower the seizure threshold in a patient with pre-existing epileptogenic networks. However, these mechanisms remain hypothetical and were not directly demonstrated in this patient.

Clinicians should maintain a high index of suspicion for acute post-infusion seizures in patients receiving donanemab, particularly those with a history of epilepsy, even in the absence of radiographically detectable ARIA. Clinicians should be aware of this potential safety signal in patients with pre-existing epilepsy. Additional studies are needed before specific monitoring or treatment recommendations can be established.

## Introduction

Donanemab (Kisunla®) is a humanized IgG1 monoclonal antibody directed against N-terminal pyroglutamate-modified forms of amyloid-beta (Aβ). The drug was recently approved by the United States (US) Food and Drug Administration (FDA) for the treatment of early symptomatic Alzheimer’s disease (AD) [1]. While donanemab significantly reduces amyloid plaque burden and mitigates cognitive decline, its pharmacological profile necessitates vigilant monitoring for amyloid-related imaging abnormalities (ARIA). This includes, but is not limited to, edema (ARIA-E) and microhemorrhage or superficial siderosis (ARIA-H) [1].

Seizures are a recognized, albeit uncommon, adverse effect of anti-amyloid immunotherapies. In data from randomized trials and subsequent pharmacovigilance reports, seizures and status epilepticus have almost exclusively been described as sequelae of severe ARIA [1]. Patients with AD exhibit a substantially higher baseline risk of unprovoked seizures compared to age-matched cognitively normal individuals. These risks are further compounded by comorbid epilepsy [2,3]. Consequently, current appropriate use recommendations for donanemab advise caution or exclusion of candidates with a history of seizures [1].

To the best of our knowledge, published case reports disentangling the direct epileptogenic potential of anti-amyloid antibodies from ARIA-mediated structural disruption are rare. Here, we present what we believe to be the first documented case of recurrent, temporally linked, non-ARIA-associated generalized seizures following donanemab infusion in a patient with established focal epilepsy.

## Case presentation

A 90-year-old man with mild cognitive impairment and AD pathology and a history of focal epilepsy was previously well-controlled on levetiracetam 500 mg twice daily. He presented for his fifth monthly donanemab infusion overall, corresponding to his second maintenance-dose infusion of 1,400 mg administered intravenously. The infusion concluded uneventfully at 11:27 AM. Approximately 45 minutes post-infusion, the patient developed a generalized convulsive episode lasting two to four minutes, characterized by unresponsiveness, jaw clenching, and bilateral upper extremity clonic movements, followed by a prolonged period of post-ictal confusion.

Emergency department evaluation revealed stable vital signs and an unremarkable comprehensive metabolic panel as well as a complete blood count. An emergent non-contrast brain MRI performed on a 1.5 Tesla scanner (Siemens Healthineers, Erlangen, Bavaria, Germany), including FLAIR and SWI sequences, showed no evidence of ARIA-E or ARIA-H (Figure 1).

**Figure 1:**
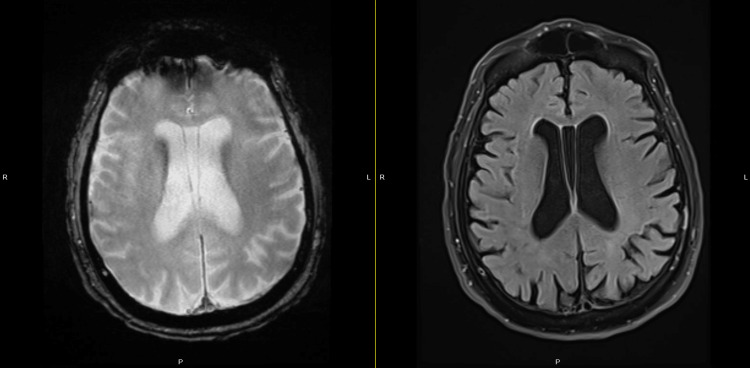
MRI of the brain Representative axial brain MRI images obtained during evaluation for amyloid-related imaging abnormalities (ARIA). Left: Susceptibility-weighted imaging/gradient recalled echo (SWI/GRE) sequence demonstrating no cerebral microhemorrhages, macrohemorrhages, or superficial siderosis, with no evidence of ARIA with hemosiderin deposition (ARIA-H). Right: Axial fluid-attenuated inversion recovery (FLAIR) sequence showing no vasogenic edema, sulcal effusions, or other abnormalities suggestive of ARIA with edema (ARIA-E).

A routine 30-minute EEG demonstrated generalized slowing consistent with diffuse cerebral dysfunction or post-ictal state, but no definitive epileptiform discharges (Figure 2).

**Figure 2:**
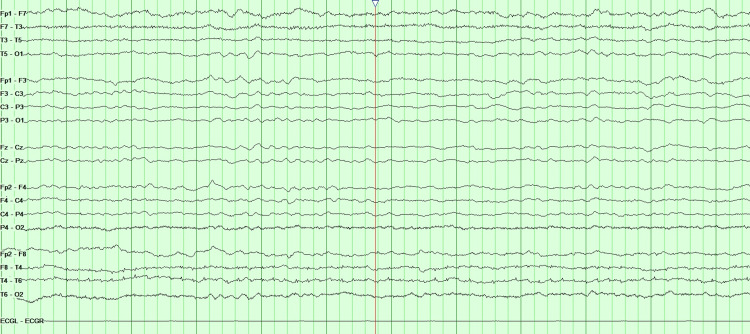
Routine EEG A routine 30-minute EEG demonstrated generalized background slowing without epileptiform discharges or electrographic seizures, consistent with diffuse cerebral dysfunction or a post-ictal state.

The event was presumptively categorized as a breakthrough seizure, and he was discharged on his baseline anti-seizure medication (ASM) regimen.

One month later, following his sixth monthly donanemab infusion overall (third maintenance-dose infusion of 1,400 mg), the patient experienced a second generalized tonic-clonic seizure within one hour of the infusion's conclusion. This episode was more prolonged, requiring emergency medical services transport and IV levetiracetam loading. Subsequent neuroimaging, including a follow-up MRI two weeks post-event, again confirmed the absence of ARIA. Donanemab therapy was permanently discontinued, and his levetiracetam dose was escalated to 750 mg twice daily. The patient remained seizure-free during the subsequent 30-day observation period. Because the initial seizure was considered a potentially provoked event temporally associated with donanemab infusion, and the patient had remained seizure-free for over one year on a stable dose of levetiracetam, no immediate changes were made to his antiseizure medication regimen after the first episode. Following recurrence after the subsequent infusion, donanemab was discontinued, and levetiracetam was increased to 750 mg twice daily.

The temporal relationship, which occurred one month later following the next infusion, as well as the absence of alternative systemic precipitants and the persistently normal neuroimaging, is compatible with a donanemab-induced lowering of the seizure threshold independent of macroscopic ARIA.

Despite being older than the population enrolled in the pivotal TRAILBLAZER-ALZ studies [4,5] and having a history of focal epilepsy, the patient otherwise fulfilled clinical and biomarker criteria for early symptomatic Alzheimer's disease. His epilepsy had remained well controlled on levetiracetam 500 mg twice daily, and he had been seizure-free for more than one year prior to initiation of donanemab therapy. Following a detailed discussion with the patient and his family regarding the potential benefits, risks, and limited available safety data in patients with pre-existing epilepsy, a shared decision was made to proceed with donanemab therapy.

## Discussion

Seizure risk in Alzheimer’s disease

The intersection of AD and epilepsy represents a complex pathophysiological convergence. Older adults with AD possess a markedly elevated risk of unprovoked seizure. This risk has been estimated to be up to 10-fold higher than that of their cognitively intact peers [3]. This heightened epileptogenicity is thought to stem from Aβ-induced neuronal hyperexcitability, potentially mediated by the disruption of inhibitory interneuron function and the unmasking of latent excitatory networks [6]. When a patient harbors a pre-existing epileptogenic focus, as in this patient, it appears that the neural circuitry is essentially "kindled," rendering it exquisitely sensitive to subtle neurochemical perturbations.

A limitation of this report is that serum levetiracetam concentrations were not measured before or during treatment; however, the patient reported adherence to therapy and had remained seizure-free for over one year prior to donanemab initiation. A further limitation of this case is that only a routine EEG was obtained. Routine EEG recordings have limited sensitivity for detecting interictal epileptiform discharges, particularly in patients with focal epilepsy. Prolonged EEG monitoring or video EEG may provide a higher diagnostic yield and could be considered in future cases to better characterize potential epileptiform activity associated with anti-amyloid therapies. Additionally, MRI examinations were performed on a 1.5 Tesla scanner. While no evidence of ARIA-E or ARIA-H was identified, higher-field-strength imaging may provide greater sensitivity for detecting subtle microhemorrhagic changes.

Hypothesized mechanisms of non-ARIA ictogenesis 

The occurrence of seizures immediately following donanemab infusion, in the strict absence of MRI-detectable ARIA, is compatible with acute, sub-radiographic mechanisms of ictogenesis. We propose the following triad of pathophysiological processes.

Aβ Clearance and Network Hyperexcitability

Preclinical models indicate that the rapid disaggregation and removal of extracellular Aβ can transiently alter synaptic homeostasis. Aβ oligomers are known to interact with N-methyl-D-aspartate (NMDA) receptors; their abrupt clearance may lead to a rebound enhancement of glutamatergic transmission, precipitating synchronous epileptiform discharges in vulnerable hippocampal and cortical networks [6].

Microglial Activation and Neuroinflammation

Donanemab, an IgG1 antibody, robustly engages Fc-γ receptors on microglia to facilitate plaque phagocytosis. This target engagement inevitably triggers a localized neuroinflammatory cascade, releasing pro-inflammatory cytokines such as Interleukin-1β (IL-1β) and Tumor Necrosis Factor-α (TNF-α). Elevated levels of these cytokines are well-established pro-convulsants, capable of rapidly downregulating astrocytic glutamate transporters (e.g., GLT-1), thereby increasing extracellular glutamate concentrations and drastically lowering the seizure threshold [7].

Transient Blood-Brain Barrier (BBB) Dysfunction

While overt ARIA-E represents macroscopic vasogenic edema, the initial binding of antibodies to vascular amyloid can induce microscopic, transient increases in BBB permeability. This sub-radiographic BBB dysfunction permits the extravasation of serum proteins (e.g., albumin) into the cerebral parenchyma, a process known to induce astrocytic transforming growth factor-β (TGF-β) signaling, which is highly epileptogenic [8].

Clinical implications

This case underscores critical considerations for the administration of anti-amyloid therapies in patients with comorbid epilepsy. The current appropriate use recommendations emphasize the exclusion of patients with a high risk of ARIA-related complications, noting that seizures are typically a manifestation of severe ARIA [1]. In addition, our observations suggest that a history of epilepsy constitutes an independent risk factor for acute post-infusion seizures.

Clinicians should engage in rigorous risk stratification and shared decision-making with patients and their caregivers, explicitly discussing the potential for seizure exacerbation independent of ARIA. Pre-infusion optimization, including the verification of therapeutic ASM levels and prophylactic dose adjustments, should be strongly considered. Furthermore, given the acute temporal onset observed in this case, implementing an extended post-infusion observation period (e.g., two to three hours) for high-risk patients may be a prudent safeguard.

## Conclusions

Recurrent, acute post-infusion seizures can occur following donanemab administration in patients with pre-existing epilepsy, even in the complete absence of radiographic ARIA. This phenomenon is likely driven by acute neuroinflammatory responses, transient BBB permeability, and rapid shifts in synaptic excitability. As the clinical utilization of anti-amyloid monoclonal antibodies expands, further prospective pharmacovigilance as well as mechanistic studies will help to quantify the true risk of non-ARIA seizures and to refine safety protocols for patients with comorbid epileptogenic disorders.
